# Identifying course characteristics associated with sociodemographic variation in enrollments across 159 online courses from 20 institutions

**DOI:** 10.1371/journal.pone.0239766

**Published:** 2020-10-14

**Authors:** René F. Kizilcec, Anna Kambhampaty

**Affiliations:** Department of Information Science, Cornell University, Ithaca, New York, United States of America; University of South Australia, AUSTRALIA

## Abstract

Millions of people worldwide use online learning for post-secondary education and professional development, but participation from historically underrepresented groups remains low. Their choices to enroll in online courses can be influenced by course features that signal anticipated success and belonging, which motivates research to identify features associated with sociodemographic variation in enrollments. This pre-registered field study of 1.4 million enrollments in 159 online courses across 20 institutions identifies features that predict enrollment patterns in terms of age, gender, educational attainment, and socioeconomic status. Among forty visual and verbal features, course discipline, stated requirements, and presence of gender cues emerge as significant predictors of enrollment, while instructor skin color, linguistic style of course descriptions, prestige markers, and references to diversity do not predict who enrolls. This suggests strategic changes to how courses are presented to improve diversity and inclusion in online education.

## Introduction

Human capital development in higher and continuing education relies increasingly on online learning. Total enrollment at intuitions of higher education in the United States (US) is declining whilst more and more people turn to online education for post-secondary degrees and professional development [1]. Fifteen percent of US college students are currently enrolled in fully online degree programs, and a third of on-campus students take at least one online course during their studies [2]. Worldwide online learning is poised to expand access to higher and continuing education, especially in underserved communities [3–6]. However, there is mounting evidence of online courses perpetuating historical participation and achievement gaps, including the underrepresentation of people with lower socioeconomic status and women in STEM disciplines [7–11]. Researchers have argued that the presence of visual and verbal cues in online environments can activate psychological stereotypes and reduce the sense of belonging in the learning environment for certain groups of students [12–16]. In particular, enrollment pages that provide descriptions and contextual information about courses are known to impact enrollment decisions [16, 17] and students’ eventual course grades [18]. An important unknown, however, is which course features contribute to sociodemographic variation in enrollment. Identifying influential features can provide instructors and online learning providers with a lever to adjust enrollment patterns and encourage participation from members of traditionally underrepresented groups.

Prior research has studied specific multimedia cues expected to raise identity-based concerns and influence anticipated belonging, including gender-stereotypic TV commercials [19, 20], promotional videos for STEM events [21], artifacts in computer science classrooms [16, 22, 23], and the design of a course website [14]. Their findings confirm that visual and verbal cues can shape perceptions about the diversity climate and reduce anticipated belonging and success. Fewer studies have demonstrated that these cues also affect real-world behaviors. One study found social advertising with images and messages to encourage women to take a STEM course to raise female enrollment [12]. Another study found a diversity statement on the enrollment page to raise enrollment among people with low educational attainment but not enrollment among women or people in developing countries [17]. Two more studies found the gender of people in lecture videos to influence women’s engagement inside of the course [13, 15]. These four studies offer causal evidence that certain visual and verbal cues can impact course participation for some sociodemographic groups. However, to achieve a more holistic understanding of major cues and their relation to who enrolls, we need a comprehensive and systematic analysis of which common course features do and do not predict sociodemographic variation in participation.

We conducted a large-scale field study to identify important features on the enrollment pages of 159 courses from 20 international institutions on two major online platforms. Online course enrollment pages have relatively standardized features across platforms. This enables us to investigate the following research question at large: Which features of course enrollment pages predict enrollment patterns in terms of age, gender, educational attainment, and socioeconomic status? We focus on these four social groups that have been identified in prior work as underrepresented in online learning: women (especially in STEM subjects), people with low educational attainment, people in developing countries, and older (mid- to late-career) learners [24–27]. We investigate sociodemographic variation along these four dimensions among 1.4 million enrollments across a wide range of courses and institutions. We study this variation as a function of different course features, because features can impact enrollments, but the causal arrow rarely points the other way.

The current research combines a pre-registered confirmatory study with an exploratory analysis and a replication study. First, using a taxonomy of psychologically inclusive design cues [12], we coded numerous features on the enrollment pages of all available courses on the Stanford Online platform. After examining the empirical distributions of these features, we pre-registered hypotheses about their relationship with specific enrollment patterns (e.g., Courses with female instructors have higher rates of female enrollment). We obtained official course enrollment numbers for each sociodemographic group under investigation to test our hypotheses and explore unexpected patterns. Finally, to replicate our findings in a new environment, we sampled over one hundred online courses offered by twenty international institutions on edX, a major online education platform. We coded their enrollment pages for features that were important in the first sample and tested which patterns replicate with official enrollment data. The findings of this research highlight course features that warrant close attention by policy-makers and researchers in efforts to broaden participation, as well as course features that warrant less attention in this context.

### Hypotheses

We pre-registered theory-based hypotheses about which course features are likely to predict sociodemographic patterns in course enrollment (https://osf.io/z2g4p/). These hypotheses focus on patterns in female enrollment and enrollment from developing countries, because there is sufficient evidence to inform these hypotheses, unlike for patters in age and educational attainment. The hypotheses are organized into three tiers based on the strength of evidence in support of them: primary, secondary, and exploratory.

Our first primary hypothesis is grounded in official statistics documenting the continued underrepresentation of women and minorities in STEM fields [28]. We therefore expected lower female participation in STEM courses (H1). Grounded in a large body of work documenting the importance of academic role models [29–31], we expected instructor characteristics to influence enrollment patterns. In particular, female learners who notice a lack of female instructors on the course page may anticipate a reduced sense of belonging and decide not to enroll [32, 33] (H2). Likewise, people in developing countries who notice an absence of non-white course instructors may anticipate a reduced sense of belonging and decide not to enroll [8, 34]. We therefore expected to observe higher female participation in courses with more female instructors and higher participation from developing countries in courses with instructors that appear ethno-racially diverse (H3).

**H1** Courses in STEM subjects have lower rates of female enrollment.
**H2** Courses with (photos of) female instructors have higher rates of female enrollment.
**H3** Courses with (photos of) a majority of white instructors have lower rates of enrollment from developing countries.

Our secondary hypotheses are informed by research that has demonstrated a link between linguistic features of job advertisements and gender differences in the perceived appeal of those jobs [35, 36]. Specifically, this work found that job ads with more masculine than feminine wording caused women to think that more men worked in the occupation and lowered the job’s appeal to women. We therefore expected to observe lower female participation in courses with descriptions that have a more masculine tone (H4). Sociolinguistic research on gender differences has also found women to be more attuned to emotional language, especially negative sentiments, and to avoid negativity in their own language production [37, 38]. Course descriptions that highlight negative emotions (e.g., for courses covering war crimes, nuclear disasters) may thus reduce the appeal of the course to women. We therefore expected to observe lower female participation in courses with descriptions with a more negative sentiment (H5). Moreover, as a visual counterpart to the linguistic features, and as theorized in research on the global achievement gap in MOOCs [8], we expected lower participation from developing countries in courses that exhibit overt markers of prestige, such as pictures of august university campuses (H6).

**H4** Courses with descriptions that have more masculine tones have lower rates of female enrollment.
**H5** Courses with descriptions that have more negative sentiment analysis scores have lower rates of female enrollment.
**H6** Courses with prestige markers have lower rates of enrollment from developing countries.

Our exploratory hypotheses encode expectations with limited empirical support in the literature to date. Extending the analysis of how female participation may be related to linguistic features of course descriptions, we expected course descriptions invoking ‘joy’ to be construed as welcoming and encouraging female enrollment (H7), while descriptions invoking ‘disgust’ would be construed as unappealing and limit female enrollment (H8) [39, 40]. Next, we expected references to diversity on the course page to send a signal of inclusion to women and increase their enrollment (H9), as has been observed in prior studies [12, 41]. The remaining exploratory hypotheses concern common course properties: whether the course has prerequisites, quizzes or tests, and how many hours of weekly effort are required. Prior work has found that men are more likely to apply for a job when they meet only 60% of the stated qualifications, while women are more likely to apply when they meet all qualifications [42]. We therefore expect to observe lower female enrollment in courses with prerequisites (H10). Indications that a course contains quizzes or tests may raise identity-related threat and lower participation among women and people in developing countries (H11) [43, 44]. Finally, the estimated number of hours per week that a course requires is expected to be associated with enrollment from developing countries: a high number may discourage or intimidate people who face scarce resources, but it may also be perceived as a signal of high value [45]. We therefore expect that its association with participation can go in either direction (H12).

**H7** Courses with descriptions that express more joy have higher rates of female enrollment.
**H8** Courses with descriptions that express more disgust have lower rates of female enrollment.
**H9** Courses with references to diversity have higher rates of female enrollment.
**H10** Courses with prerequisites have lower female enrollment.
**H11** Courses with quizzes or tests have lower rates of (a) female enrollment, and (b) enrollment from people in less-developed countries.
**H12** Courses with higher weekly effort have higher (or lower) rates of enrollment from less-developed countries.

## Methods

### Course sample selection

We examined two independent samples of online courses from different platforms. The first sample (Stanford sample, *n* = 54 courses, 311,818 total enrollments) was drawn from courses offered on Stanford Online, a platform for free online courses, graduate and professional certificates, advanced degrees, and executive education programs offered by Stanford University. We considered all courses that were open for enrollment and offered in English, yielding a set of 54 courses with a wide range of subjects, enrollment sizes, and levels of difficulty. The second sample (edX sample, *n* = 105 courses, 1,090,168 total enrollments) was drawn from courses offered on edX.org, one of the largest platforms for massive open online courses, for-credit courses, and bachelor’s and master’s degree programs offered by 130 global partners (nonprofits, universities, companies, etc.) [46]. From the 2,600 courses offered on edX.org, we selected 105 courses from twenty institutions to approximate a representative sample. We restricted the list of potential courses to those offered by member institutions of the edX research data sharing program, because we could obtain official enrollment data for them. We selected a wide range of subjects from a variety of global institutions, oversampling courses from US institutions (71 out of 105), and including at least three courses from each of the major subject areas on the edX platform (Arts & Culture; Business & Management; Computer Science; Biology & Life Sciences; Energy & Earth Sciences; Engineering; Humanities; Social Sciences; etc.).

### Course feature coding

**Fig 1:**
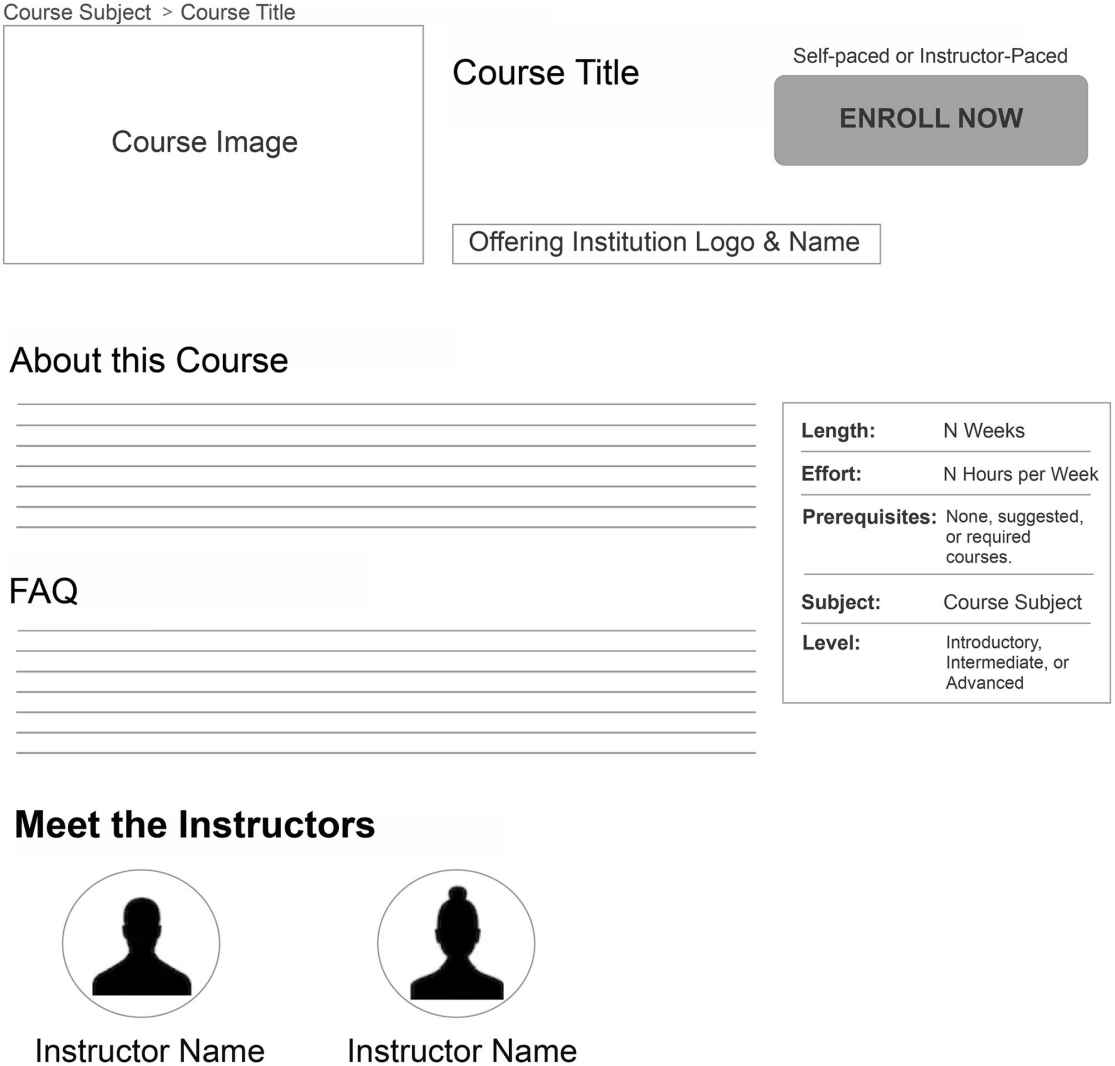
Schematic representation of a course enrollment page. A wireframe illustration of a course enrollment page highlighting key features that were coded.

The coding scheme was developed in iterations by closely examining the course enrollment pages in the Stanford sample for features that can be codified and that a potential learner sees before enrolling. Course enrollment pages on a given platform follow a standard template, as illustrated in the wireframe schematic in Fig 1. The specific layout and design varies between platforms, while the set of features remains mostly constant. Grounded in the taxonomy of psychologically inclusive design cues [12], we coded for visual content cues (e.g., photos of course instructors) and verbal content cues (e.g., presence of prerequisites, tone of text); we did not code for visual design cues because they are constant throughout a given platform, nor did we code for interaction design cues because there was no opportunity for interaction before enrolling.

Instructor gender and race were coded manually by the authors on the basis of instructor photos and names on the enrollment page. Gender was coded as -1 if all instructors were male, 0 if there were male and female instructors, and 1 if all instructors were female. Race was coded according to skin color for courses with instructor photos present as 0 if half or more instructors appeared to be white, and 1 if the majority of instructors were not white.

We used two online natural language processing tools to code linguistic features of text that appears on enrollment pages: IBM Watson’s Natural Language Understanding API for sentiment analysis (negative to positive; [-1,1]) and emotion detection (Sadness, Joy, Fear, Disgust, Anger; [0, 1]), as well as Textio Hire for an overall score (how inviting the text sounds to potential job applicants; [0, 1]; https://textio.com/) and gendered tone analysis (how much the tone appeals to masculine versus feminine applicants; [-3,3]; https://www.ibm.com/cloud/watson-natural-language-understanding). The IBM Watson tool is grounded in psycholinguistic research and widely used; it applies a stacked generalization-based ensemble method with features including n-grams, punctuation, greetings, and sentiment polarity to classify emotion categories. For standard emotion data sets such as ISEAR and SEMEVAL, IBM Watson’s emotional tone analysis achieves statistically better average performance than the best accuracy of state-of-the-art models: average F1 score of 41% and 68% compared to 37% and 63%, respectively (https://cloud.ibm.com/docs/tone-analyzer). The Textio Hire tool uses a proprietary algorithm trained on thousands of job descriptions and applicant information to predict general appeal and gender bias. We checked its face validity in the present context by sampling course page texts with high and low Textio scores, which confirmed a stark contrast in language: the masculine text included words like ‘business’, ‘companies’, ‘trade’, ‘leadership’, ‘top executives’, while the feminine text included words like ‘empathy’, ‘trust’, ‘happiness’, ‘emotional and social intelligence’.

The coding scheme for each sample is shown in Table 1; a detailed code book and data for the Stanford sample can be found in our pre-registration document (https://osf.io/z2g4p/). The coding scheme for the edX sample was based on the results for the Stanford sample. We coded features in the edX sample that showed a robust correlation (defined as *p*- value < 0.1 and *r* > 0.25) with at least one enrollment measure in the Stanford sample. We excluded linguistic features for the FAQ text as few courses in the edX sample presented a separate FAQ text. We additionally coded five exploratory features: three features that we pre-registered hypotheses about (Instructor Race, Intro in Course Title, and “About” Text Textio Tone) and two new features to codify institutional variation, since edX courses were offered by various international institutions (the institution’s 2019 QS World University ranking and the 2018 Human Development Index, or HDI, of the country where the institution is located; https://www.topuniversities.com/university-rankings/world-university-rankings/2019).

**Table 1:** Coded features of course enrollment pages in both samples organized by the psychologically inclusive design taxonomy [12].

Taxonomy	Stanford Sample	edX Sample
Visual Cues	Instructor Gender, Instructor Race, Promo Photo Gender, Promo Photo Race, Prestige Markers, Reference to Diversity	Instructor Gender, Instructor Race
Verbal Cues	Course Title, Course Subject, STEM Subject, Course Level, Intro in Course Title, Prerequisites, Weekly Effort, Quizzes or Tests, Self-paced, Reference to Diversity,“About” Text *(Watson Sentiment, Sadness, Joy, Fear, Disgust, Anger; Textio Score, Tone)*,“Requirements” Text *(Watson Sentiment, Sadness, Joy, Fear, Disgust, Anger; Textio Score, Tone)*,“FAQ” Text *(Watson Sentiment, Sadness, Joy, Fear, Disgust, Anger; Textio Score, Tone)*	STEM Subject, Prerequisites, Weekly Effort, Intro in Course Title,“About” Text *(Watson Sadness; Textio Score, Tone)*
Other	—	Institution Country HDI, Institution Ranking

### Measures of enrollment

We use four indicators of sociodemographic enrollment patterns that were established in prior research [17]: (1) the percentage of female learners in the course, (2) the percentage of learners who reside in countries with a United Nations Human Development Index (HDI) below 0.7 (officially classified as low-to-medium development), (3) the percentage of learners who do not have a college degree, and (4) the average age of learners. We obtained course-level aggregate enrollment data for each of the courses in both samples after we pre-registered our hypotheses. Gender, age, and education level was self-reported by learners during the enrollment process; HDI was determined using the country-level location from the IP address during enrollment.

### Analytical approach

After coding the course pages in the Stanford sample, we examined the empirical distributions of all coded features to focus our attention on those with sufficient variance to find robust correlations with enrollment patterns. Our pre-registered hypotheses (https://osf.io/z2g4p/) were informed in part by the distributional characteristics of these variables, summarized in S1 Table. For the pre-registered analysis, we computed Pearson correlation coefficients and corresponding p-values using the *cor.test()* function in *R*. We used Pearson correlation coefficients, because they can be computed consistently for course features that are binary, ordinal, or continuous; they can be compared directly across outcomes and features, and their interpretation is intuitive to most readers. We first tested the correlates as specified in the hypotheses and then expanded our analysis to examine correlations between other coded variables and enrollment patterns. Finally, for course characteristics that show significant associations with enrollments across both samples, we estimate the overall strength of the association in terms of group averages. To this end, we fit a linear regression model predicting the measure of enrollment (e.g., percentage of female enrollment) with one course characteristic (e.g., a binary indicator for course prerequisites) and a mean-centered platform fixed effect to account for platform differences. Test statistics are computed using robust standard errors to account for potential heteroskedasticity.

### Data and code availability

**Fig 2:**
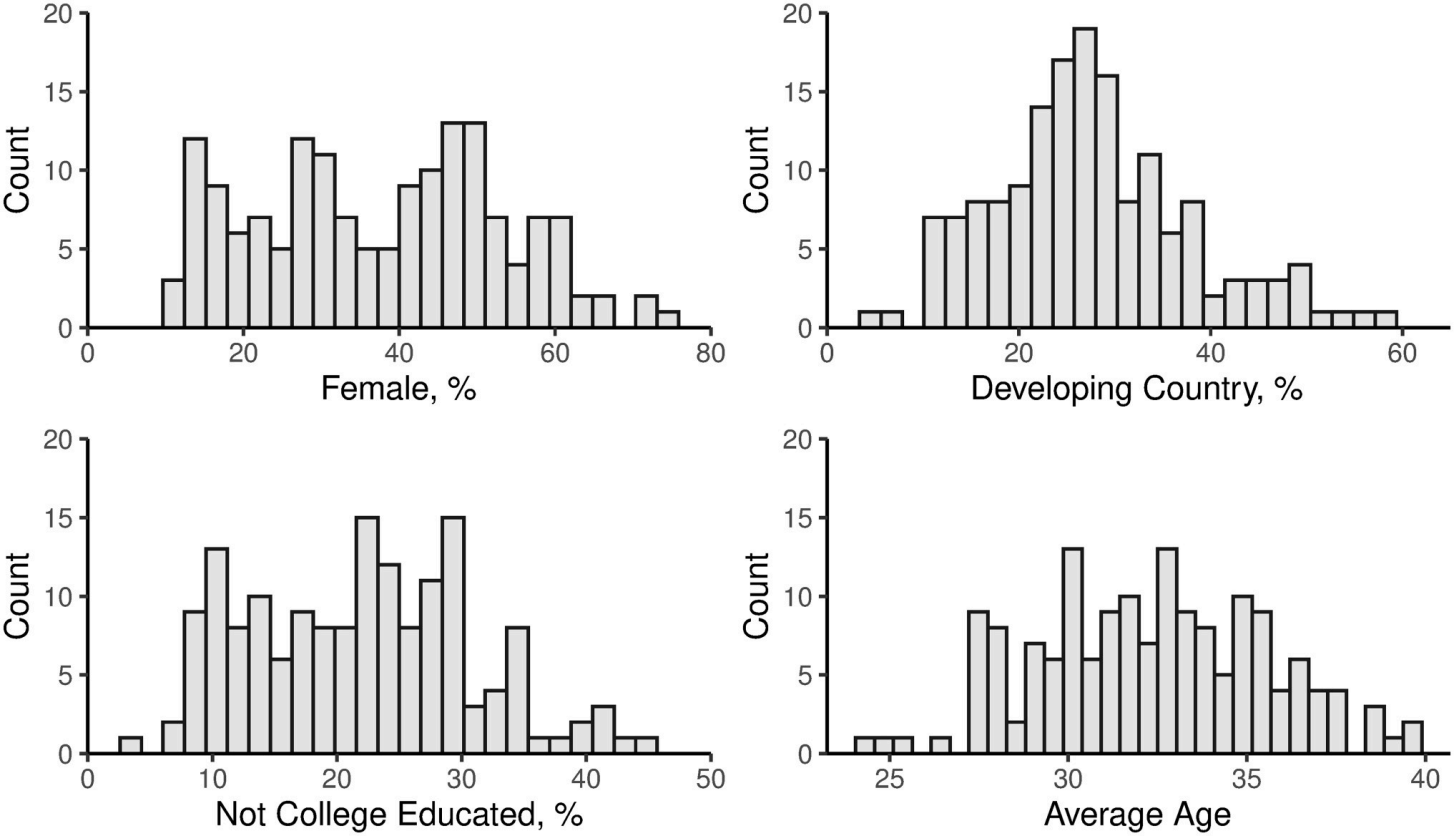
Course-level distributions of sociodemographic enrollment outcomes. Histograms showing the distribution of each measure of enrollment for the 159 courses in the Stanford and edX sample. The sample mean (and standard deviation) for each measure are 38% (16%) female, 28% (10%) from developing countries, 22% (8.9%) not college educated, and 32 (3.2) average age.

**Fig 3:**
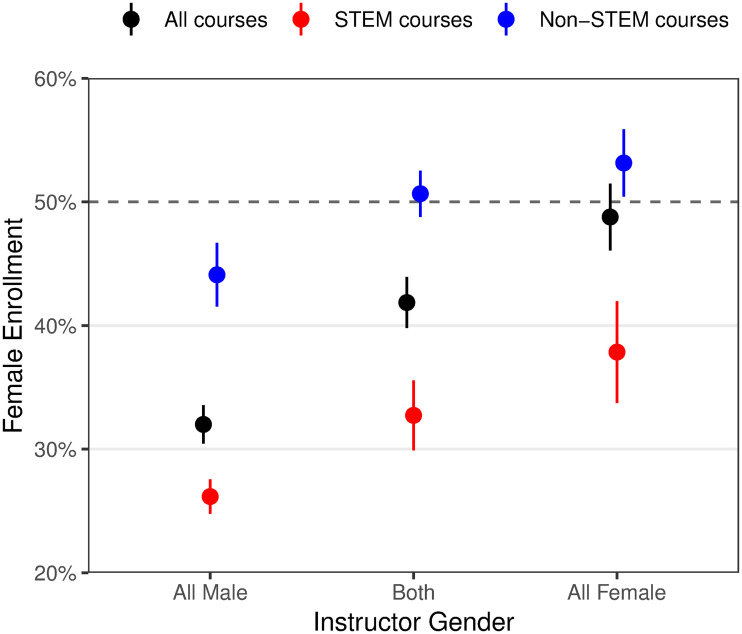
Female enrollment by instructor gender and course discipline. Showing the average enrollment rate in STEM courses (red), non-STEM courses (blue), and all combined (black); error bars are 1 SEM.

**Fig 4:**
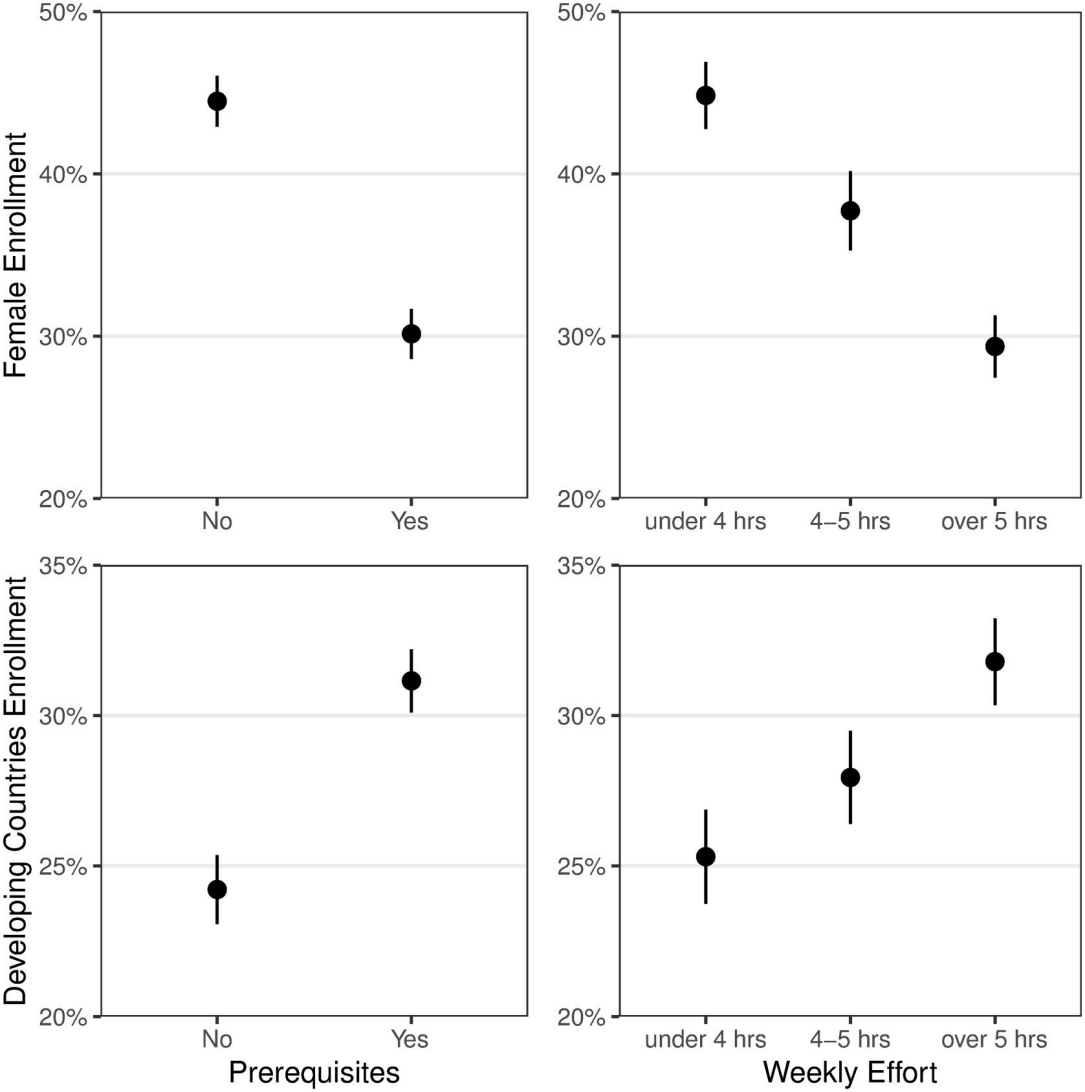
Female and low-HDI enrollment by prerequisites and weekly effort. Female enrollment (top panels) and enrollment from developing countries (bottom) varies with whether a course specifies prerequisites (left) and its expected amount of effort (right). Error bars show 1 SEM.

Raw data for both samples and analysis code for reproducing Figs 2–4 are available at https://osf.io/z2g4p/.

## Results

Sociodemographic enrollment patterns vary substantially across the 159 courses, as shown in Fig 2. The average course enrollment is 38% female, 22% not college educated, 28% from developing countries (HDI < 0.7), and 32 years in age. The substantial spread in these distributions enables us to identify predictive course features: across all courses female enrollment ranges from 10% to 75%, developing country enrollment from 5% to 59%, not college educated enrollment from 4% to 45%, and average age from 24 to 40. Courses with higher female enrollment tended to enroll older students (Pearson correlation: *r* = 0.37, *p* < 0.001) and fewer from developing countries (*r* = −0.63, *p* < 0.001), but with similar college education levels (*r* = −0.05, *p* = 0.54). Courses with lower college educated enrollment tended to enroll younger students (*r* = −0.52, *p* < 0.001) and more from developing countries (*r* = 0.29, *p* < 0.001). Courses with higher developing country enrollment also enrolled younger students (*r* = −0.43, *p* < 0.001).

Results are presented in chronological order, beginning with confirmatory and exploratory findings for the Stanford sample (Table 2), then findings for the edX sample (Table 3), and finally a summary of findings that replicate across both samples (Table 4).

**Table 2:** Confirmatory and exploratory findings in the Stanford sample.

Coded Features	Female	Developing Country	Not College Educated	Average Age
*Primary*				
STEM Subject	-0.609***	0.255*	0.141	-0.458***
Instructor Gender	0.570***	-0.414***	0.004	0.150
Instructor Race	0.073	-0.061	-0.067	0.026
*Secondary*				
Textio Tone, About	-0.025	-0.074	-0.216	-0.008
Watson Sentiment, About	-0.037	0.162	-0.066	0.113
Prestige Markers	-0.196	0.092	-0.150	0.232*
*Exploratory*				
Watson Joy, About	-0.033	0.093	-0.005	0.153
Reference to Diversity	0.196	-0.045	0.052	0.108
Prerequisites	-0.471***	0.109	-0.190	0.046
Weekly Effort	-0.625***	0.215	0.010	-0.335*
Quizzes or Tests	0.070	-0.089	0.140	0.064
Watson Disgust, About	0.084	0.088	0.173	-0.023
*Post-hoc*				
Watson Sadness, About	0.060	-0.072	0.459***	-0.306**
Textio Score, About	0.350***	-0.083	0.017	0.295**
Textio Score, FAQ	0.288**	-0.122	-0.157	0.214
Intro in Course Title	-0.144	0.172	0.222	-0.191
Self-paced	0.204	-0.036	0.149	0.006

Pearson correlations between primary, secondary, exploratory and post-hoc features and sociodemographic enrollment variables. Absolute correlations above 0.25 in bold font.

*** *p* < 0.01,

** *p* < 0.05,

* *p* < 0.10

**Table 3:** Confirmatory and exploratory findings in the edX sample.

Coded Features	Female	Developing Country	Not College Educated	Average Age
*Confirmatory*				
STEM Subject	-0.631***	0.503***	0.030	-0.272***
Instructor Gender	0.346***	-0.109	0.088	0.054
Prerequisites	-0.506***	0.421***	-0.305***	-0.093
Weekly Effort	-0.339***	0.288***	0.029	-0.238**
Watson Sadness, About	0.229**	-0.147	0.127	-0.071
Textio Score, About	0.073	0.046	0.297***	-0.181
*Exploratory*				
Instructor Race	0.059	-0.133	-0.199**	0.117
Intro in Course Title	-0.116	0.194**	0.221**	-0.221**
Textio Tone, About	0.264***	-0.076	-0.0804	0.111
Institution Ranking	-0.055	-0.038	0.119	0.101
Institution Country HDI	-0.231**	0.230**	-0.167*	0.192**

Pearson correlations between coded features and sociodemographic enrollment variables.

*** *p* < 0.01,

** *p* < 0.05,

* *p* < 0.10

**Table 4:** Summary of significant associations with enrollment in both samples.

Association	Difference in Enrollment	Test Statistics
*Female Enrollment*		
STEM Subject	29% STEM vs. 49% non-STEM	*t*_156_ = 10.0, *p* < 0.001
Instructor Gender	32% male vs. 41% both vs. 50% female	*F*_2,155_ = 15.2, *p* < 0.001
Weekly Effort	31% for 7hrs vs. 44% for 2.5hrs	*t*_136_ = 5.79, *p* < 0.001
Prerequisites	29% with vs. 45% without	*t*_156_ = 7.13, *p* < 0.001
*Developing Country Enrollment*		
STEM Subject	31% STEM vs. 22% non-STEM	*t*_156_ = 5.93, *p* < 0.001
*Average Enrollment Age*		
STEM Subject	31 STEM vs. 33 non-STEM	*t*_156_ = 4.45, *p* < 0.001
Weekly Effort	31 for 7hrs vs. 33 for 2.5hrs	*t*_136_ = 3.42, *p* = 0.001

Estimates derived from linear regression models with robust standard errors and course platform fixed effect. For Weekly Effort, 7 and 2.5 hours represent 1 standard deviation above/below the mean.

### Findings in the Stanford sample

There is support for two of the three primary hypotheses in the Stanford sample: STEM courses have lower rates of female enrollment (H1) and courses with more female instructors have higher rates of female enrollment (H2). However, the hypothesized relationship between instructors’ ethno-racial diversity and enrollment from developing countries is not supported (H3). None of our secondary hypotheses concerning linguistic features and the presence of prestige markers are supported either (H4-6). Among the exploratory hypotheses (H7-12), we only find evidence that courses with prerequisites have lower rates of female enrollment (H10). Corresponding correlation coefficients with levels of statistical significance are presented in Table 2.

Beyond our hypotheses, we discover a number of unexpected significant associations between pre-registered course features and enrollment measures. STEM courses and courses with more male instructors have a higher rate of enrollment from developing countries. STEM courses, courses without prestige markers, and courses demanding more weekly effort enroll younger people. Courses demanding more weekly effort also have a lower rate of female enrollment. In subsequent post-hoc analyses of the remaining course features, we also find that certain linguistic features—sadness and appealing tone—significantly correlate with sociodemographic patterns in enrollment.

### Findings in the edX sample

We conducted confirmatory analyses in the edX sample for six course features that exhibited a robust association (*p* <.05 and *r* >.25) with enrollment outcomes in the Stanford sample. We find strong evidence that these features are also predictive of enrollment patterns in this broader sample (Table 3). STEM courses again have lower rates of female enrollment (H1), a higher rate of enrollment from developing countries, and enroll younger people. Likewise, courses with more female instructors have a higher rate of female enrollment (H2). Courses with prerequisites have lower rates of female enrollment (H10), but higher rates of enrollment from developing countries and college-educated people. Courses demanding more effort have lower rates of female enrollment, but higher enrollment from developing countries (H12) and younger people. Courses with sad descriptions enroll more women in the edX sample, though in the Stanford sample, they enroll younger, less educated people. While the correlation between Textio Score for FAQs and female enrollment is robust in the Stanford sample, we are unable to replicate the finding because most courses in the edX sample do not have FAQs.

In addition to the confirmatory analyses, we explore correlations for three of the pre-registered features plus two institution-level features since the edX sample contains courses from 19 international institutions. We find that courses with (a majority of) non-white instructor(s) have higher enrollment among college-educated people. Courses with titles indicating an introductory level have higher rates of enrollment from younger people, those without a college degree, and those in developing countries. Courses with descriptions that use a feminine tone have higher rates of female enrollment (H4). Although the world ranking of the course institution does not predict sociodemographic enrollment patterns, its geographic location does. Specifically, courses offered by institutions in more developed countries have a higher rate of enrollment from males, people in developing countries, and older, college-educated people.

### Consistent findings across samples

A number of course features stand out as significant predictors of sociodemographic enrollment patterns across both samples. They are summarized in Table 4.

Women account for only 29% of enrollments in STEM courses compared to 49% in non-STEM courses (H1). The percentage of women is higher in courses with more female instructors (H2): 32% all male instructors, 41% both male and female instructors, and 50% all female instructors. Fig 3 visualizes this remarkably linear trend, which appears for STEM and non-STEM courses alike. That is, even in courses covering non-STEM subjects, more women enroll if there are more female instructors.

More women enroll in courses without any prerequisites (45% without vs. 29% with; H10) and courses that demand less weekly effort: the average percentage of women is 44% in a course demanding 2.5 hours compared to 31% in a course demanding 7 hours. These associations are illustrated in Fig 4 (top row). STEM courses are twice as likely to have prerequisites than non-STEM courses (60% vs. 33%; *χ*^2^ = 10.1, *p* = 0.001) and demand 1.2 hours, or 29%, more weekly effort on average (*t*_137_ = 3.24, *p* = 0.001). Yet even within the subset of STEM courses, having prerequisites and demanding more weekly effort predicts lower female enrollment simultaneously in a regression model (prerequisites: *β* = −0.10, *t*_73_ = −3.06, *p* = 0.003; *z*-scored effort: *β* = −0.024, *t*_73_ = −2.30, *p* = 0.024). The pattern is the same for non-STEM courses (prerequisites: *β* = −0.089, *t*_58_ = −2.85, *p* = 0.006; *z*-scored effort: *β* = −0.045, *t*_58_ = −2.66, *p* = 0.010).

Overall, 57% of variation in the proportion of female enrollment can be explained by just four course characteristics: STEM subject, instructor gender, weekly effort, and prerequisites (multiple regression *R*^2^ = 0.566 with platform fixed effect; *F*_6,132_ = 28.7, *p* < 0.001). Despite correlations between these course characteristics, each one is a significant predictor of female enrollment in a combined ANOVA (each *p* < 0.001).

Enrollment from developing countries also varies based on course prerequisites and weekly effort (Fig 4, bottom row). This pattern is significant in the edX sample but not in the (smaller) Stanford sample, and therefore does not appear in Table 4. Nevertheless, across both samples, developing country enrollment is significantly higher in courses with prerequisites (31% with vs. 24% without; *t*_156_ = 4.33, *p* < 0.001) and in courses demanding more weekly effort (48% for 7hrs vs. 35% for 2.5hrs; *t*_136_ = 3.15, *p* = 0.002). Developing country enrollment is also higher in STEM courses (see Table 4), which are more likely to have prerequisites and demand more weekly effort. Repeating the analysis separately for STEM and non-STEM courses, we find that for non-STEM courses having prerequisites and demanding more weekly effort predicts higher developing country enrollment simultaneously in a regression model (prerequisites: *β* = 0.70, *t*_58_ = 3.35, *p* = 0.001; *z*-scored effort: *β* = 0.017, *t*_58_ = 2.01, *p* = 0.049). However, the associations are attenuated and not statistically significant among STEM courses (prerequisites: *β* = 0.017, *t*_73_ = 0.58, *p* = 0.56; *z*-scored effort: *β* = 0.015, *t*_73_ = 1.20, *p* = 0.24).

Overall, 24% of variation in the proportion of developing country enrollment can be explained by just three course characteristics: STEM subject, weekly effort, and prerequisites (multiple regression *R*^2^ = 0.238 with platform fixed effect; *F*_4,134_ = 10.4, *p* < 0.001). Despite correlations between these course characteristics, each one is a significant predictor of developing country enrollment in a combined ANOVA (each *p* < 0.025).

Finally, we find that STEM courses and courses demanding more weekly effort attract younger audiences on average (Table 4). The negative association between age and weekly effort is significant for STEM courses (*β* = −0.676, *t*_74_ = −2.43, *p* = 0.017), but attenuated for non-STEM courses (*β* = −0.444, *t*_59_ = −1.36, *p* = 0.179). Overall, 30% of variation in average enrollment age can be explained by just two course characteristics: STEM subject and weekly effort (multiple regression *R*^2^ = 0.303 with platform fixed effect; *F*_4,134_ = 10.4, *p* < 0.001; each *p* < 0.015).

## Discussion

Efforts to increase the participation of historically underrepresented populations in educational programs, such as women in STEM fields, have been underway for decades [28]. Numerous studies have documented how concerns about anticipated belonging and a “chilly climate” can cause people to opt for different courses and academic pathways [16, 22, 23, 47]. Much prior work has studied how manipulating a particular feature in an educational environment, such as classroom decorations [22], affects students’ attitudes towards the environment. More recent work has extended this paradigm to investigate the behavioral consequences of specific course features in the field, such as how a diversity statement or social cues in course videos affect online course participation [13, 15, 17, 48]. These research efforts have produced causal evidence that visual and verbal cues can influence student attitudes and behavior, but they have skipped over answering a broader research question with significant implications: how are commonly used features of course pages related to course participation patterns?

To answer this question, we conducted a comprehensive and systematic investigation of how course features are related to sociodemographic variation in enrollment. We combined official enrollment records for 159 courses across 20 institutions from two major course platforms with expert coding of course features based on a taxonomy of psychologically inclusive design [12]. Unlike prior work in this area, we consider variation in four population characteristics—age, gender, educational attainment, national development context—to gain a holistic view of enrollment patterns. We also consider a much larger number of features simultaneously to distinguish ones that matter from those that do not. Our results confirm some theory-based hypotheses about important features but not others, and highlight the relative importance of currently underappreciated features. We find that 57% of variability in female enrollment across courses is explained by four basic course features: course subject, instructor gender, and signals of academic rigor. We now discuss how our behavioral findings contribute large-scale empirical evidence to psychological theories of identity and motivation, inspire new research directions, and provide practical recommendations for policy-makers.

### The role of the course subject

The subject area of a course stands out as a key predictor of who enrolls. We find strong and robust evidence that STEM subjects enroll more men, younger people, and more people from developing countries. We can compare this result to national studies that have documented disparities in who participates and persists in STEM fields in the context of traditional educational institutions, such as in undergraduate and postgraduate programs [28]. The gender gap we find is consistent with prior expectation and evidence. Although the low barrier to entry for these free online courses has been expected to lower sociocultural hurdles to participation, we still find women to be underrepresented in STEM courses. Improving female enrollment in STEM courses has been the subject of some experimental research in online courses with mixed success [12, 13, 15, 17]. In light of persistent stereotypes about STEM and identity-based motivations for course selection [33, 49], our results suggest that how a course topic is labeled can have profound consequences for female enrollment. Whether presenting technical courses in a way that reduces their association with STEM could improve female participation warrants further investigation.

The appeal of STEM courses among younger generations and people in developing countries may reflect the increased demand for STEM education in developing economies including China, India, and Russia that are facing shortages of qualified STEM instructors [6, 50]. This might lead more students and young professionals in developing regions to seek out STEM learning opportunities in particular.

### The role of instructor characteristics

Economists of education and social psychologists have argued that students benefit from exposure to in-group instructors, which is partly because they can act as role models to students and influence their judgements of anticipated belonging and success in the environment [29–31]. The present findings extend this insight by showing that female students are also more likely to enroll in courses with more female instructors, no matter if the course is about a STEM subject or not. Although it seems natural to focus on women when the average gender ratio in enrollments is skewed towards men, our correlational finding can also be interpreted to show that fewer men enroll in courses with more female instructors. Whether instructor gender influences either women’s or men’s or both their decisions to enroll warrants further examination. A study that manipulated the salience of instructor gender at the start of a STEM course found that women were exclusively impacted by the intervention [13], which suggests that a focus on female enrollment rates is warranted.

Instructor gender could matter primarily as a visual cue but also as a verbal cue when students infer gender by name. While all of the courses in the edX sample showed photos of instructors, 18.5% of the courses in the Stanford sample did not show instructor photos, but their gender was plausibly evident from their names. The strength of the association between instructor gender and female enrollment is similarly strong on course pages with and without instructor photos. The correlations presented in Table 2 account for courses with instructor photos, following our pre-registration. Of the ten courses without photos, seven have all male instructors and three both male and female instructors. Imputing these values yields similar correlations. Female: *r* = .515 (*p* < 0.001); Developing Country: *r* = −.382 (*p* = 0.004); Not College Educated: *r* = .078 (*p* = 0.57); Average Age: *r* = .078 (*p* = 0.58). Fig 3 shows estimates with instructor gender imputed. This finding that the correlation between instructor gender and female enrollment does not depend on the presence of photos suggests that even in the absence of a visual gender cue, people actively infer gender by name to use in their enrollment decision-making process. Policy-makers who seek to improve female participation in online courses should consider featuring more women in the instructional team on the course page to influence judgements of anticipated belonging. It may not be necessary for them to be course instructors; featuring female teaching assistants or instructional designers, which some courses already do, may also produce the desired outcome.

Prior work in higher education has argued that the benefits of identity-matched instructors also extend to underrepresented ethno-racial minorities [30, 31]. In the absence of information about ethno-racial identity for instructors or students, we instead focus on a dimension of national identity. Building on research in the context of hiring decisions that revealed patterns of discrimination against foreign-born job applicants [51], we expected a higher rate of enrollment from developing countries in courses with instructors of color. However, we did not find robust evidence that this course feature predicts any variation in who enrolls. This may be due to the coarse operationalization of instructors of color by classifying photos as white versus non-white, and of student identity by a national indicator of development.

### The role of signals of academic rigor

A surprising finding is the importance of both course prerequisites and the expected weekly effort for female enrollment and enrollment from developing countries. These two course features can be interpreted as signals of rigor that shape judgements of anticipated success in the course, even though they are notably subjective. Unlike in a traditional college class, the number of hours someone spends on the learning materials will vary substantially with their prior knowledge and experience, motivation, and learning strategies. Likewise, course prerequisites are neither enforced nor presented in a standardized manner; it is left up to instructors to decide whether to list any prerequisites. The subjective nature of these features provides instructors and platform providers with a strategic lever considering these features’ strong association with female and international enrollment. How much this lever affects enrollment and whether it encourages underprepared students to enroll warrants careful investigation. This study focuses on participation rates, not success rates, but changes in course composition may affect success rates in the aggregate.

Based on prior evidence that women have a stronger preference to meet stated qualifications compared to men [42], we hypothesized that women would be less inclined to enroll in courses with prerequisites. The same argument can be applied to the stated amount of expected weekly effort. Women might give stronger consideration than men to whether they can actually fit the stated number of hours into their weekly schedule before deciding to enroll. Notably, the association between these signals of rigor and female enrollment are observed in STEM and non-STEM courses alike. To what extent lowering the stated number of hours and de-emphasizing course prerequisites to influence judgements of anticipated success can raise female enrollment (and/or lower male enrollment) warrants further research.

The presence of prerequisites and higher weekly effort is equally predictive of enrollment from developing countries. People may interpret them as signals of rigor and quality of instruction, especially in contexts like China, India, and Russia where qualified STEM instructors are in high demand [50]. Moreover, a higher stated amount of weekly effort and course titles indicating an introductory level are features that also attract younger people, who may have more time as well as workforce-related incentives to spend on learning new topics.

Another key signal of rigor is tied to the institution that offers a course. The most commonly used measure of an institution’s prestige and rigor is its world ranking. The edX sample ranges from institutions ranked worldwide in the top 20 to ones ranked below 200, and yet we do not find any association between rank and enrollment patterns. Instead, we find the expected associations with enrollment patterns when considering the level of national development for the institution’s location. Although most of the courses are offered by universities in highly developed countries, the sample includes institutions ranging from a Human Development Index of 0.72 to 0.92. The significant albeit modest associations we observe between this measure and all four enrollment patterns suggest that potential learners are attentive to which courses are offered by institutions in wealthy countries. In particular, wealthier institutions attract higher enrollment from developing countries but lower female enrollment, mirroring the pattern for other signals of rigor.

### The role of linguistic style

Linguistic style of the text presented on course pages, especially the course description, was expected to matter for enrollment patterns. We encoded linguistic style using both sentiment and tone analysis. We find mixed evidence for their role in enrollment decisions. Our hypothesis that course descriptions written in a tone that is more appealing to women will encourage more women to enroll is supported in the edX sample but not the Stanford sample. This may be because there is more variation in tone in the edX sample, which includes courses from twenty different institutions. However, although variation in tone is actually lower in the Stanford sample, the same is true of the overall appeal score, which still significantly correlates with female enrollment in the Stanford sample but not in the edX sample. Findings of the sentiment analysis are mixed as well and course description sentiment is largely unrelated to enrollment patters, except for a mixed set of significant associations with the degree of sadness.

Although we used state-of-the-art linguistic models, this type of linguistic analysis is limited by the fact that these models tend to be proprietary and trained on datasets that do not resemble course descriptions. For platforms like edX with almost 3,000 unique courses, it may be feasible to train linguistic models using course descriptions to predict enrollment patterns and suggest edits to course staff for how to make descriptions appeal to different demographics. Insights from such a model could transfer to traditional college courses for which instructors write course descriptions with minimal guidance on how to make courses appeal to different groups of students.

## Conclusion

This research offers strong evidence that participation from historically underrepresented groups in online courses is related to the way that courses are presented. Judgements about anticipated success and belonging in the learning environment are shaped by particular course features and inform people’s decisions to enroll or not. Much is at stake given that online courses already provide 110 million people worldwide with access to post-secondary education and professional development [52]. Course platforms and instructional teams that are looking for ways to increase enrollments without perpetuating existing participation gaps will find the specific recommendations of this work valuable. The contribution of this large-scale field study is to advance our understanding of the relationship between numerous common course features and four sociodemographic enrollment characteristics. The real-world behavioral insights into which features are in fact important for enrollment decisions and which ones are not should inform the direction of future research efforts. By virtue of the study’s scale and rigorous design, including pre-registration and replication, our correlational findings provide a strong basis for studies that test the causal effects of adapting course features that are identified as important.

The evidence supports a psychological account that early cues inform judgements of anticipated belonging and success in a learning environment. Specifically, course features related to gender identity such as the instructor gender and whether it is a STEM course are known to influence perceptions of anticipated belonging [22, 32]; we show that they are also strong predictors of female enrollment. Likewise, course features related to academic rigor such as the expected effort and prerequisites are known to influence perceptions of anticipated success [42]; we show that they are strong predictors of female and international enrollment as well. Given that these course features can act as deterrents to enrollment in courses that people can join for free without prior commitment, their consequences may be amplified in educational programs that require an upfront payment to enter the course [53]. We conclude that strategic changes to course features and cover pages promise to offer a simple and cost-effective approach for improving diversity and inclusion in online education.

## Supporting information

S1 TableEmpirical distributions of primary, secondary, and exploratory features coded in the Stanford sample.Mean $\bar{x}$, standard deviation *σ*, and five-number summary *q*.(PDF)

S1 File (ZIP)
