# Supplementary material for: Identifying course characteristics associated with sociodemographic variation in enrollments across 159 online courses from 20 institutions
Source: PLoS One. 2020 Oct 14;15(10):e0239766. doi: 10.1371/journal.pone.0239766 (PMC7556443; doi:10.1371/journal.pone.0239766)
Supplement: S1 Table — Mean x¯, standard deviation σ, and five-number summary q. (PDF) [file pone.0239766.s001.pdf]

S1 Table. Empirical distributions of primary, secondary, and exploratory features coded in the Stanford sample. Mean $\overline{x}$, standard deviation σ, and five-number summary q.

**Variable**	**Distribution**
STEM Subject	33.3% yes, 66.7% no
Instructor Gender	56.8% male, 27.3% female, 16% both
Instructor Race	86.4% white, 13.6% minority presence
Prestige Markers	46.3% present, 53.7% not present
Reference to Diversity	83.3% present, 16.7% not present
Prerequisites	66.7% present, 33.3% not present
Weekly Effort	$\overline{x}$=5.1, q=[1.0, 3.0, 4.5, 7.8, 10.0]
Quizzes or Tests	61.1% present, 38.9% not present
Textio Tone, About	$\overline{x}$=0.17, σ=1.1, q=[-3.00, 0.00, 0.00, 1.00, 2.00]
Watson Sentiment, About	$\overline{x}$=0.65, σ=.24, q=[-0.35, 0.54, 0.71, 0.81, 0.98]
Watson Joy, About	$\overline{x}$=0.54, σ=.17, q=[0.16, 0.50, 0.58, 0.64, 0.79]
Watson Disgust, About	$\overline{x}$=.051, σ=.029, q=[.002, .029, .05, .067, .127]
