# Supplementary figures and images for: Identifying course characteristics associated with sociodemographic variation in enrollments across 159 online courses from 20 institutions
Source: PLoS One. 2020 Oct 14;15(10):e0239766. doi: 10.1371/journal.pone.0239766 (PMC7556443; doi:10.1371/journal.pone.0239766)

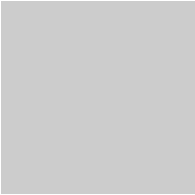

Supplement: S1 File — (ZIP) [file pone.0239766.s002.zip › figs/Fig3.pdf]

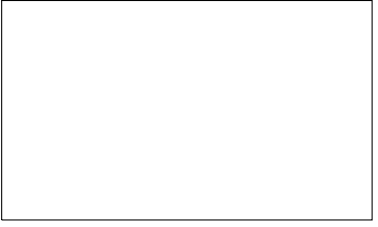

Supplement: S1 File — (ZIP) [file pone.0239766.s002.zip › figs/grabs.pdf]

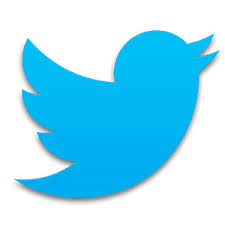

Supplement: S1 File — (ZIP) [file pone.0239766.s002.zip › thumbnails/cas-twitter.jpeg]

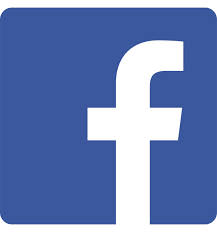

Supplement: S1 File — (ZIP) [file pone.0239766.s002.zip › thumbnails/cas-facebook.jpeg]

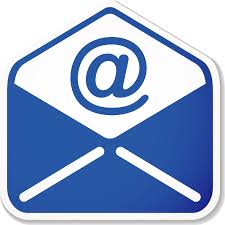

Supplement: S1 File — (ZIP) [file pone.0239766.s002.zip › thumbnails/cas-email.jpeg]

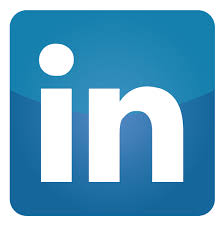

Supplement: S1 File — (ZIP) [file pone.0239766.s002.zip › thumbnails/cas-linkedin.jpeg]

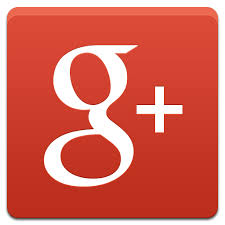

Supplement: S1 File — (ZIP) [file pone.0239766.s002.zip › thumbnails/cas-gplus.jpeg]

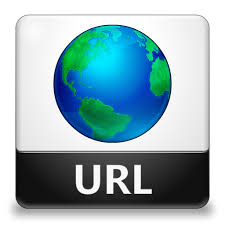

Supplement: S1 File — (ZIP) [file pone.0239766.s002.zip › thumbnails/cas-url.jpeg]

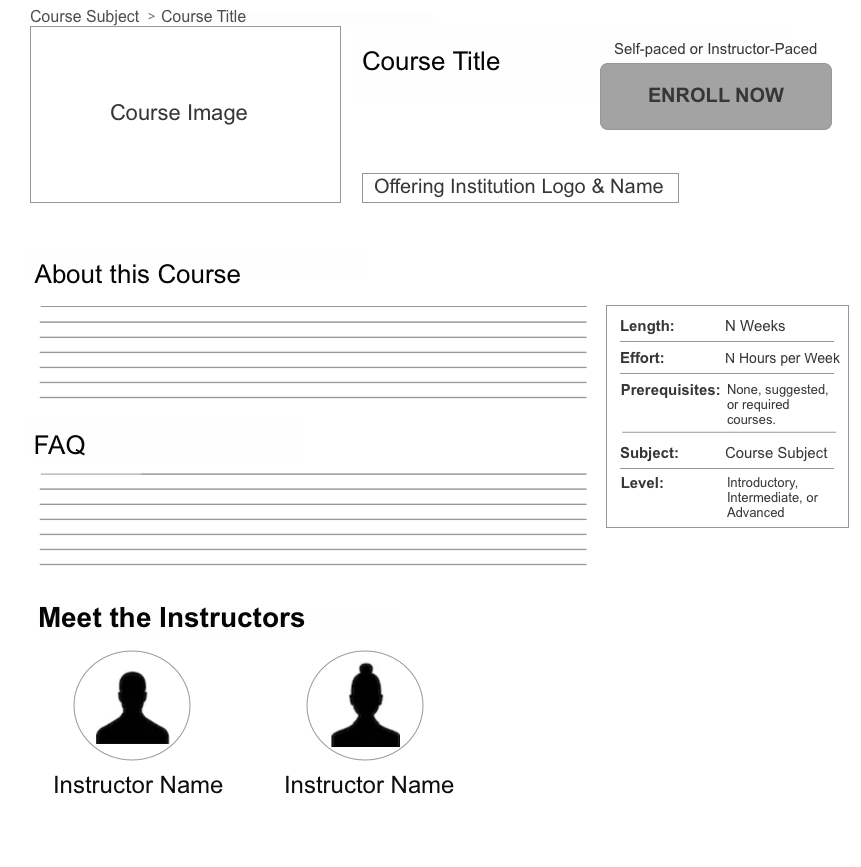

Supplement: S1 File — (ZIP) [file pone.0239766.s002.zip › img/edXWireframeResize4colourless.png]

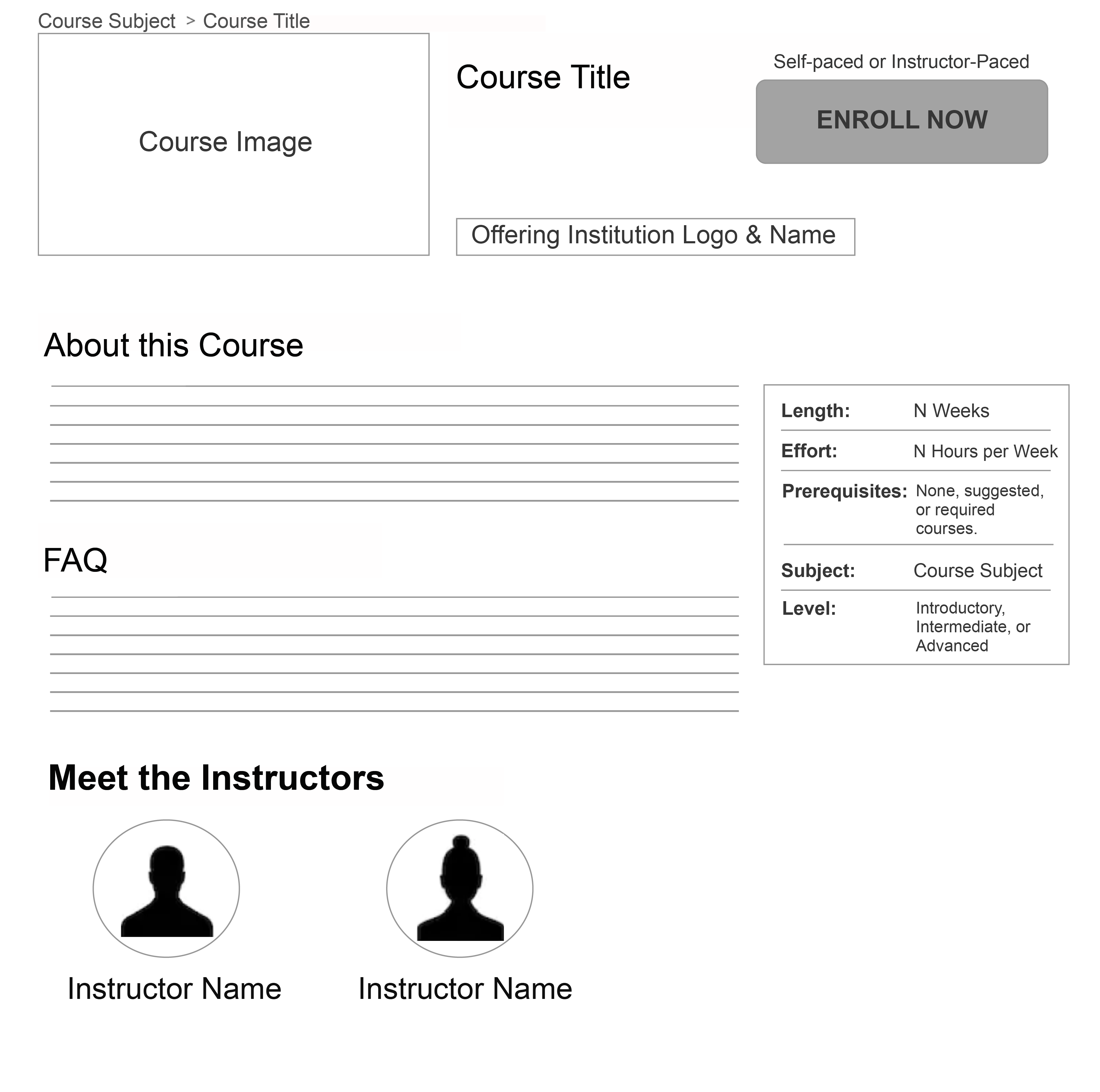

Supplement: S1 File — (ZIP) [file pone.0239766.s002.zip › img/edXWireframeHighRes.png]

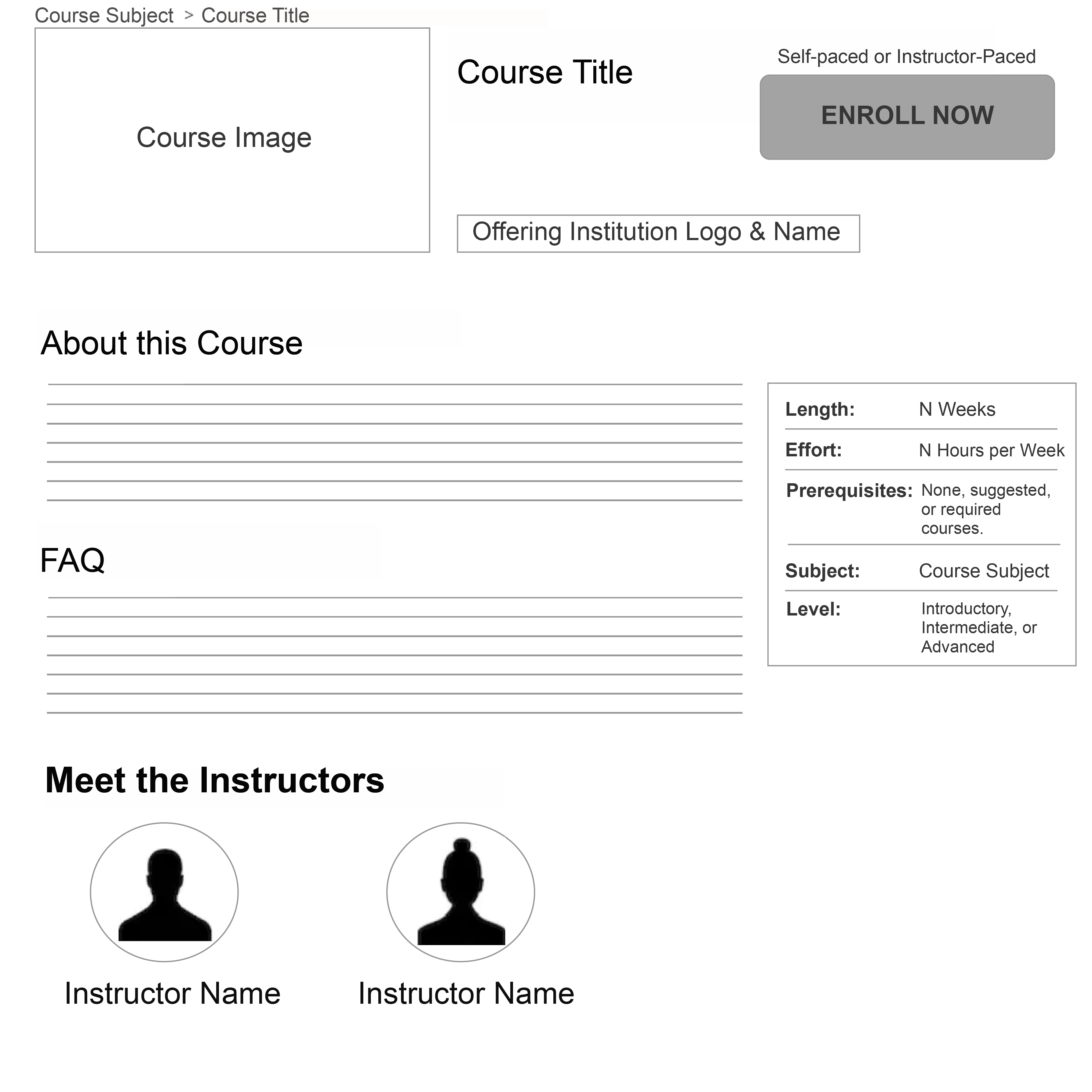

Supplement: S1 File — (ZIP) [file pone.0239766.s002.zip › img/Fig1_big.tif]

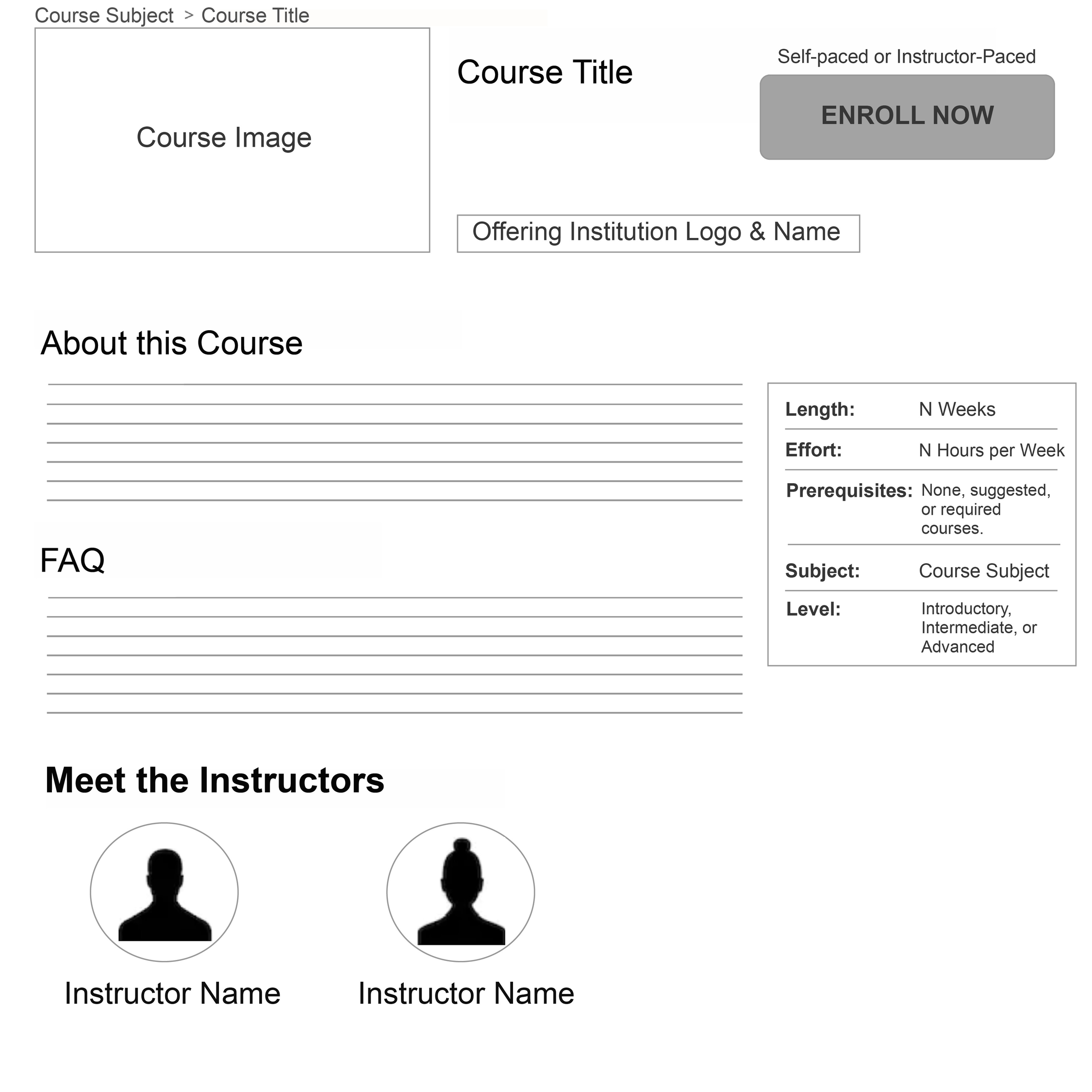

Supplement: S1 File — (ZIP) [file pone.0239766.s002.zip › img/Fig1.tif]

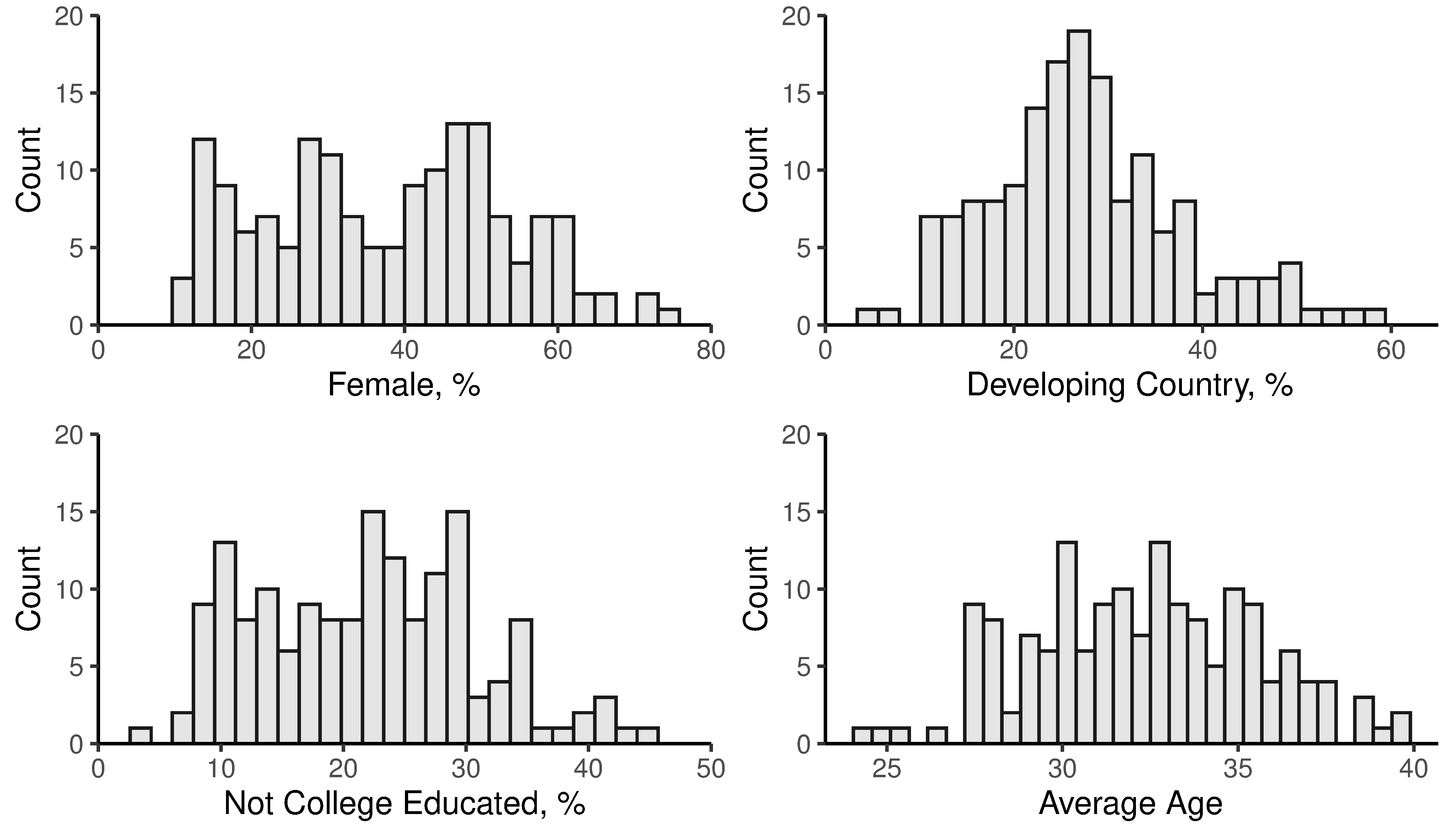

Supplement: S1 File — (ZIP) [file pone.0239766.s002.zip › img/Fig2.tif]

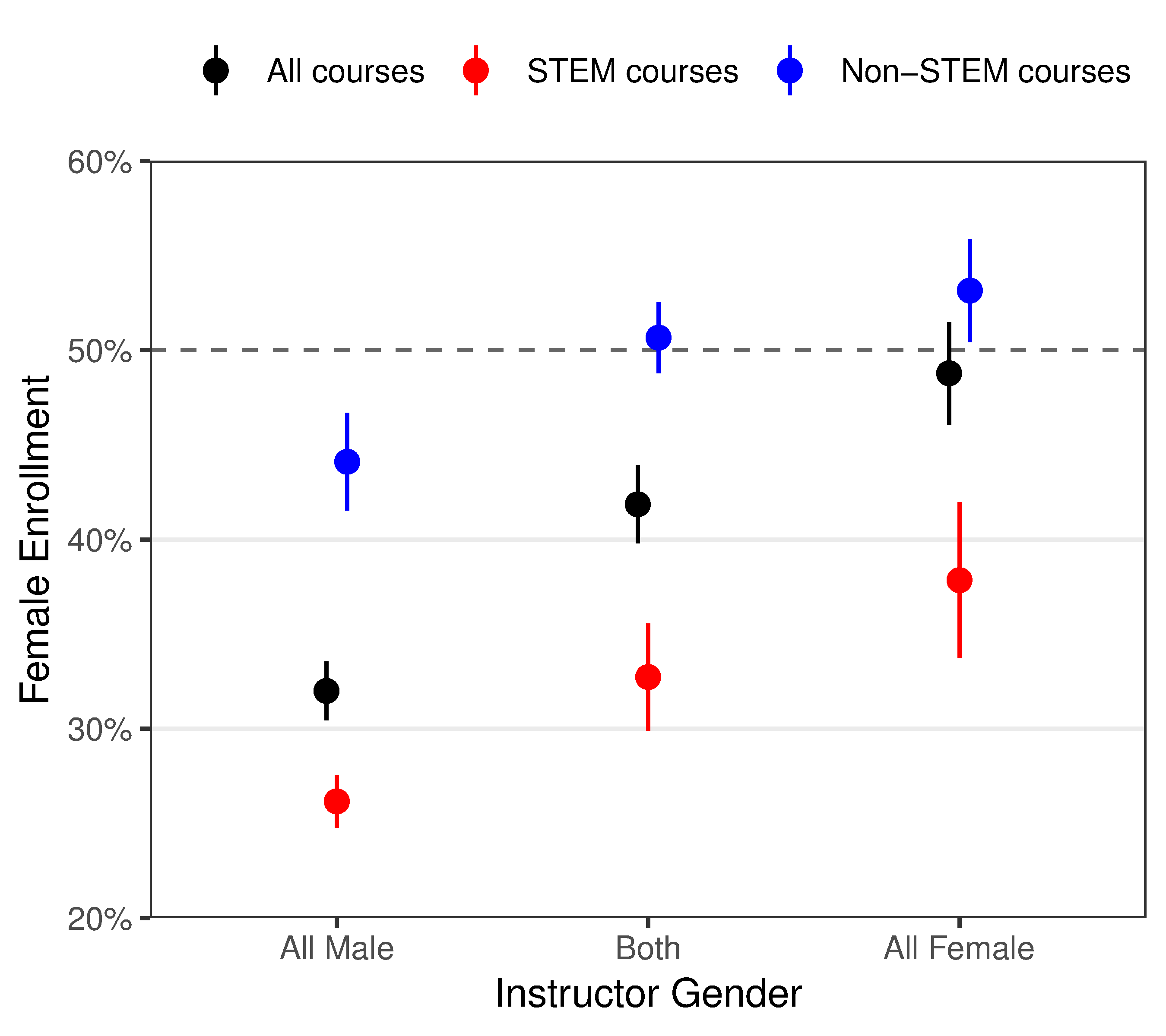

Supplement: S1 File — (ZIP) [file pone.0239766.s002.zip › img/Fig3.tif]

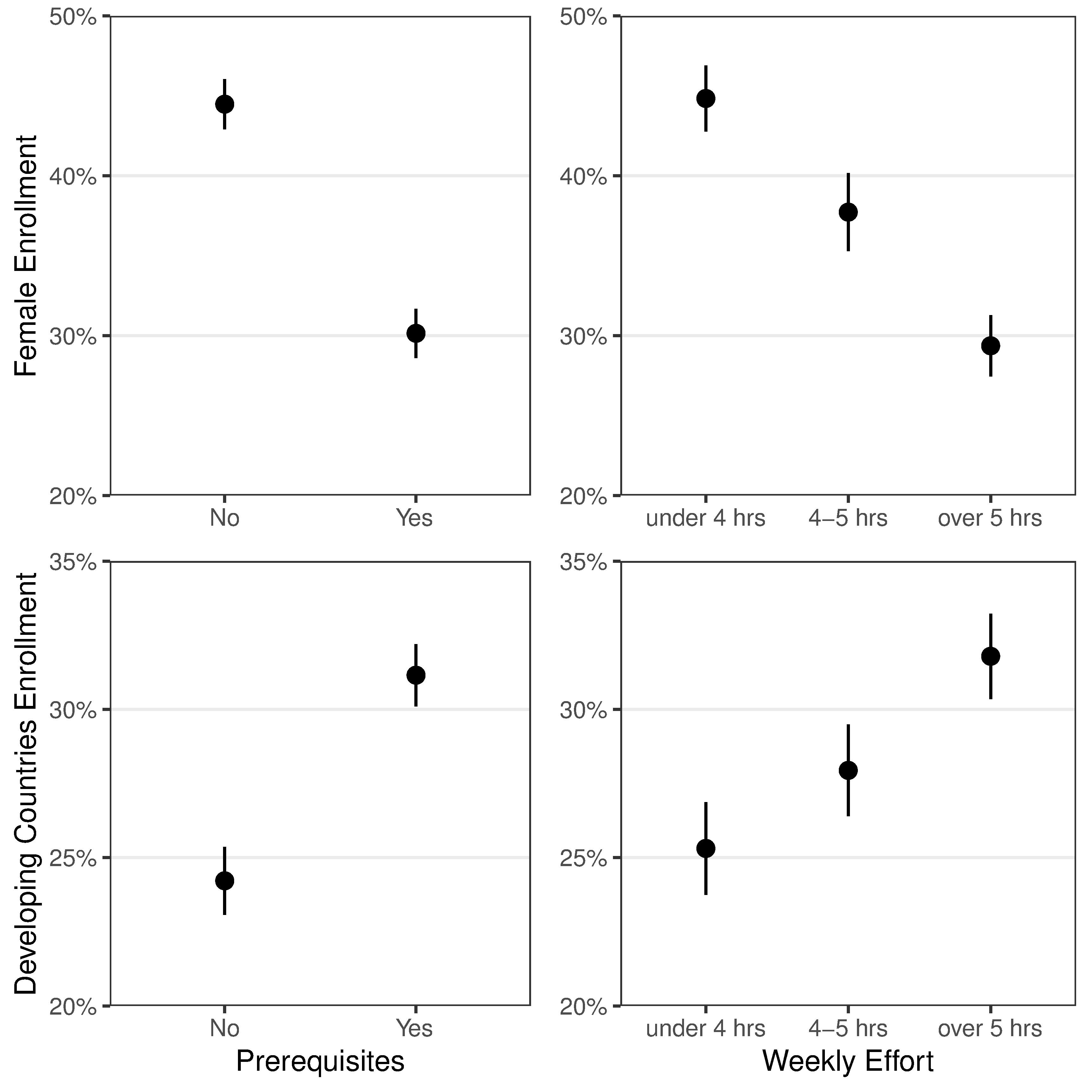

Supplement: S1 File — (ZIP) [file pone.0239766.s002.zip › img/Fig4.tif]
